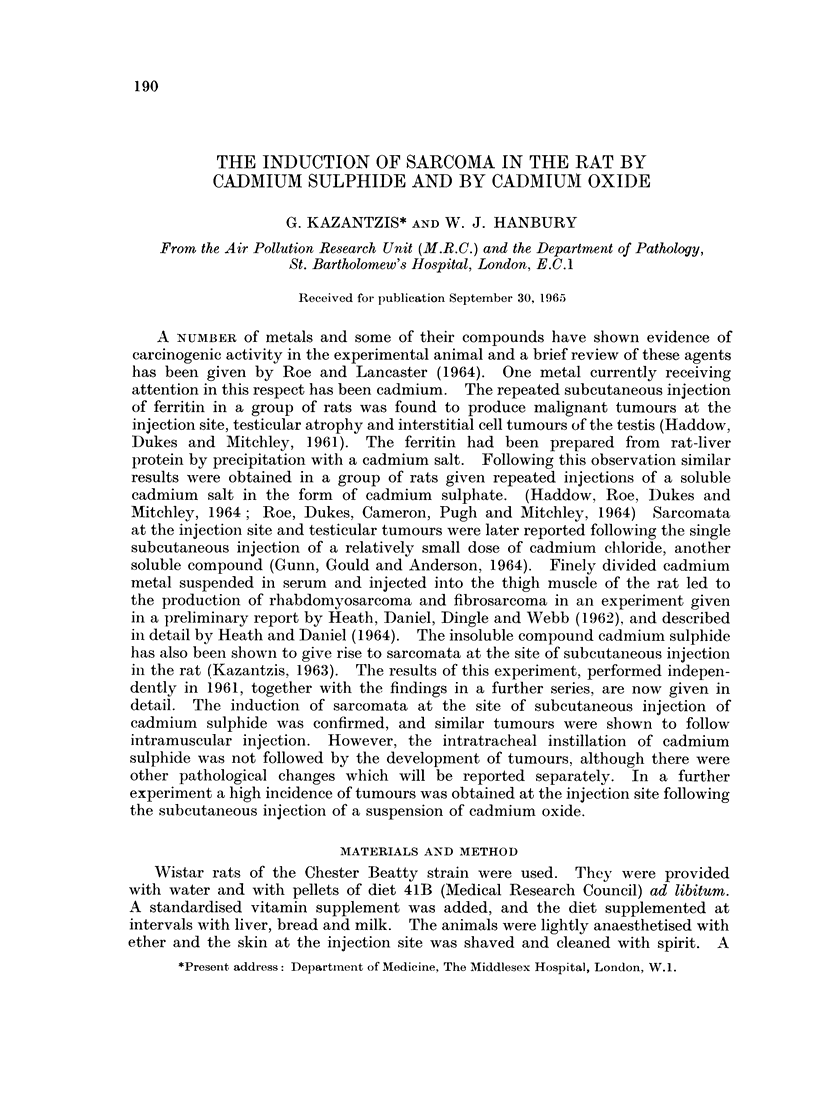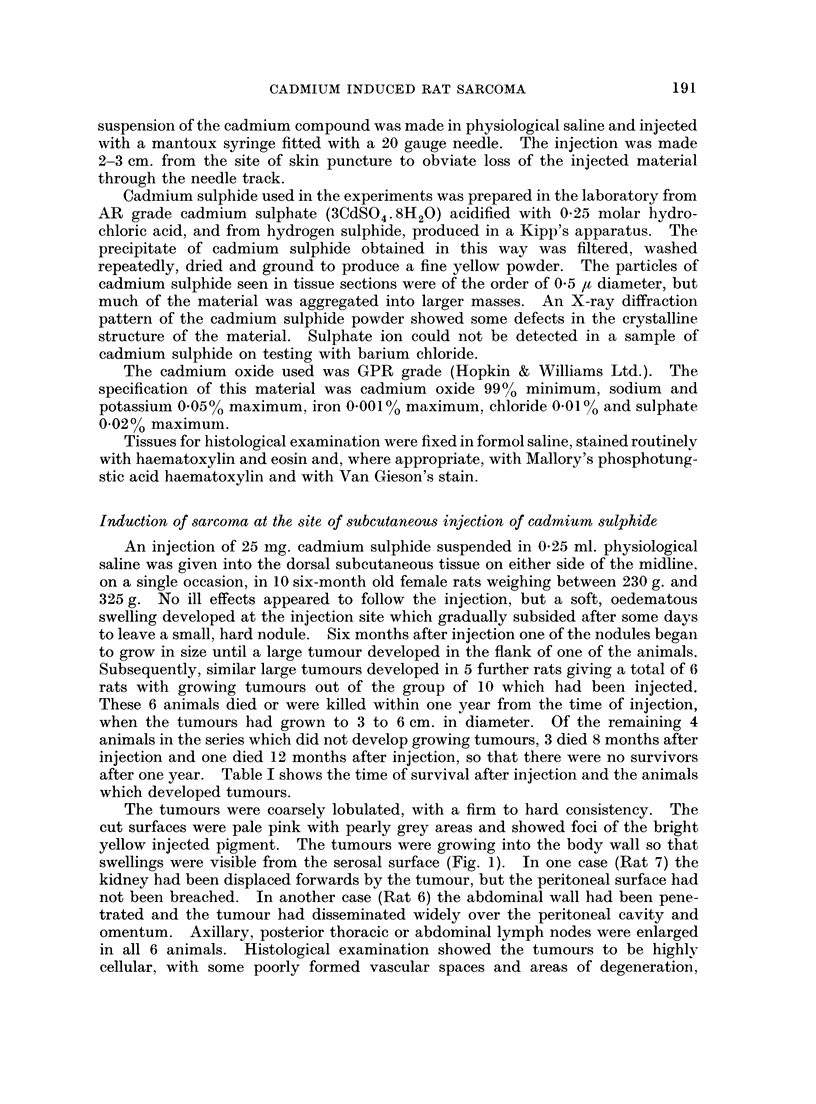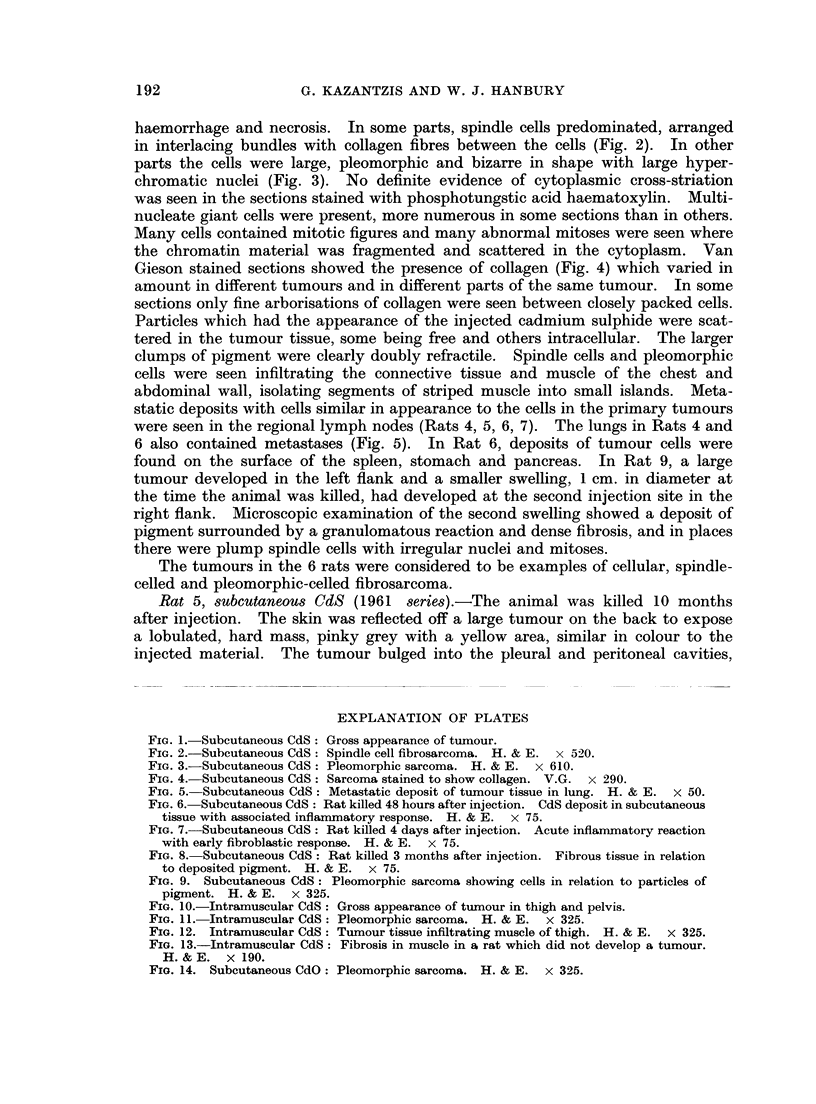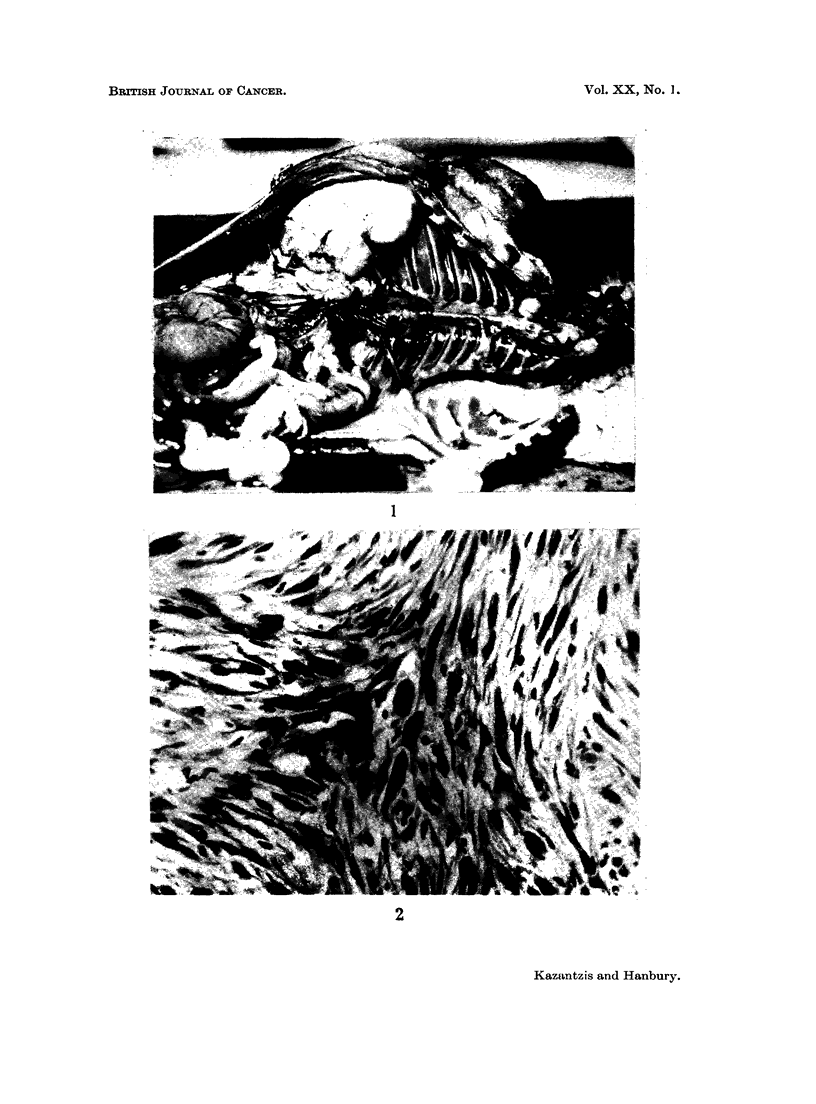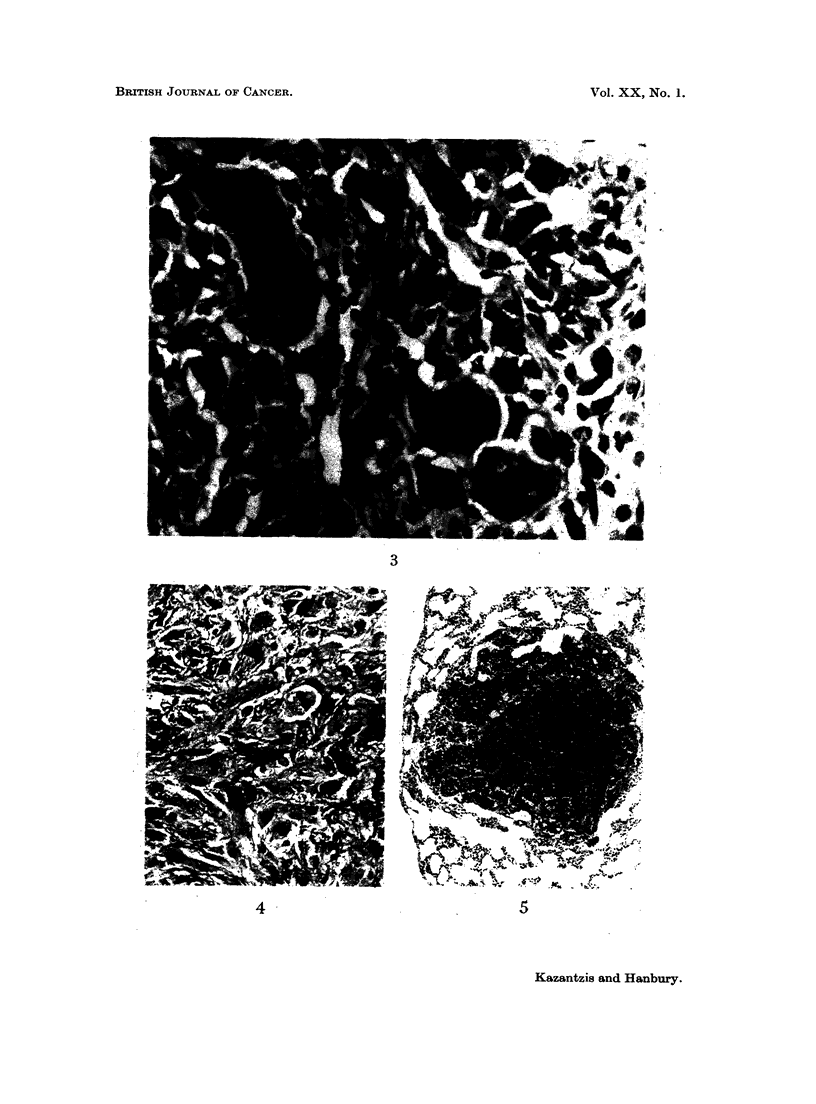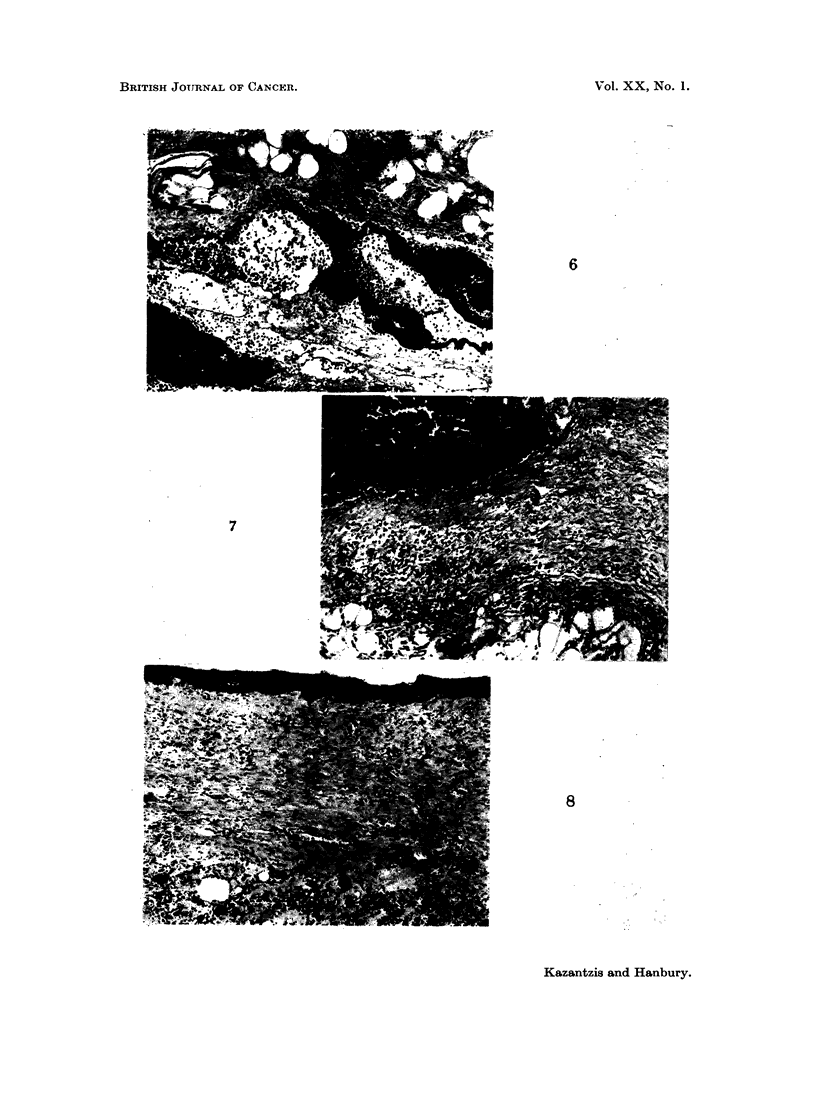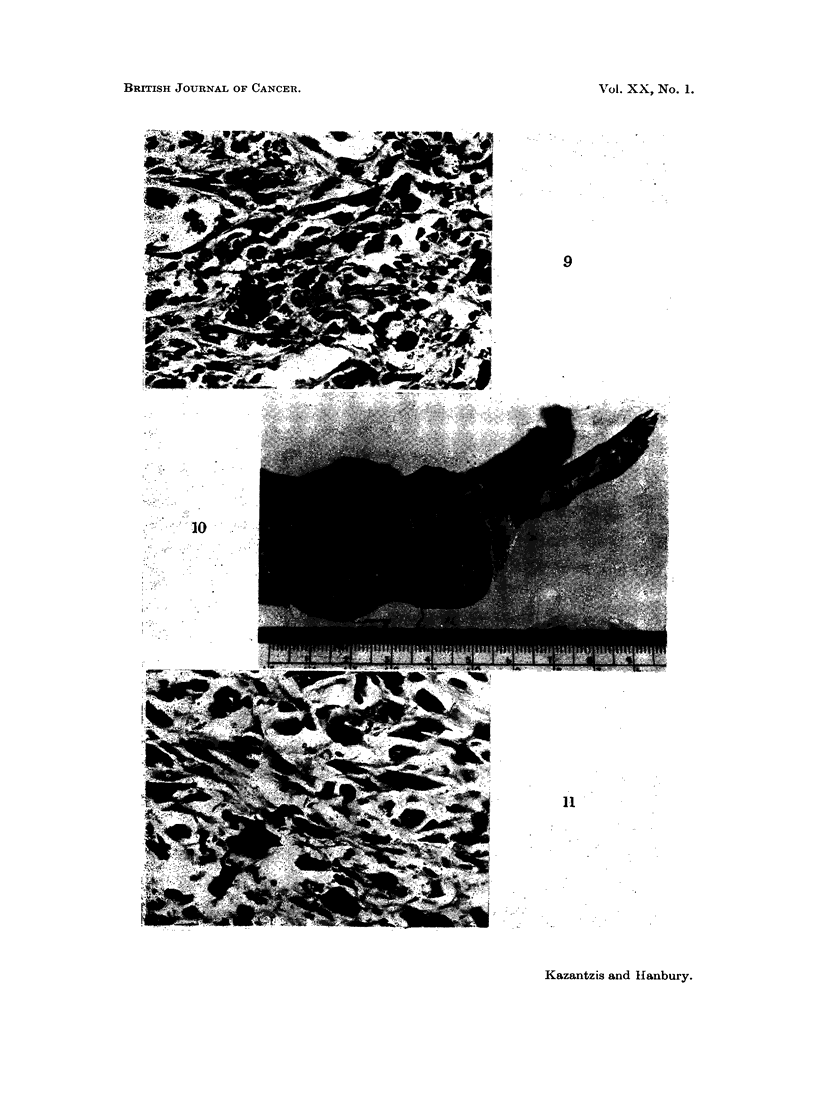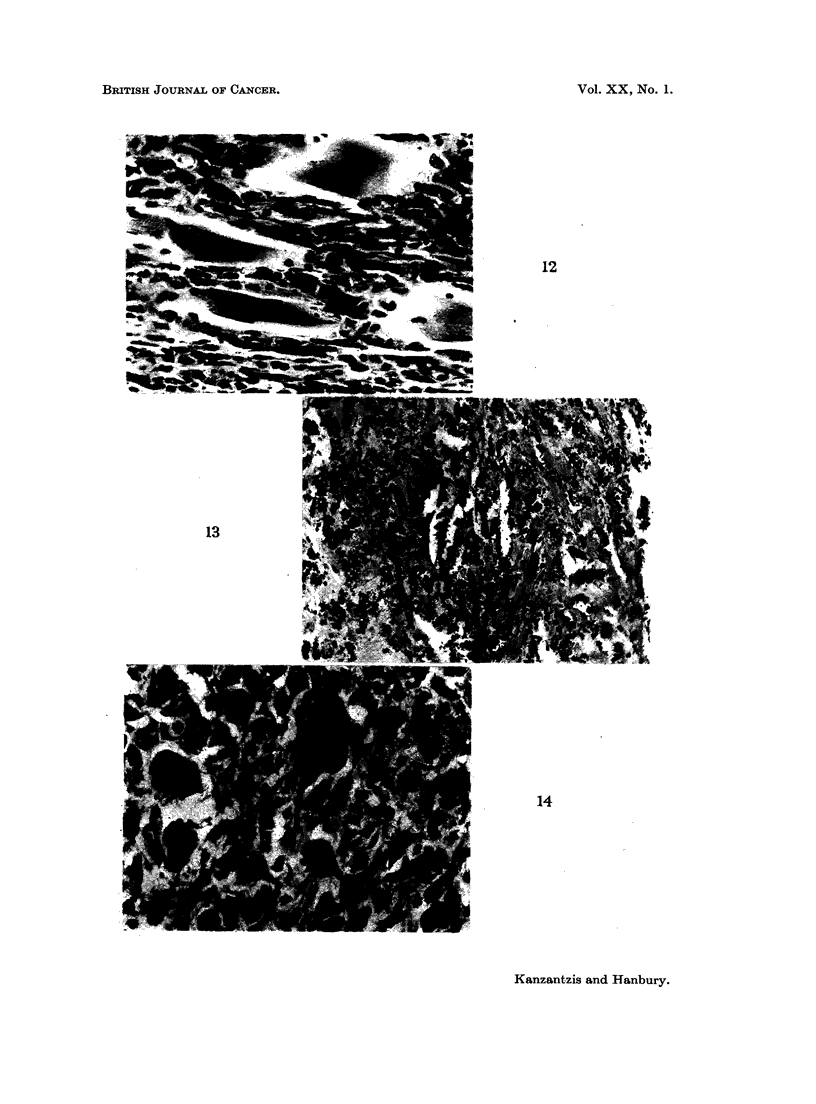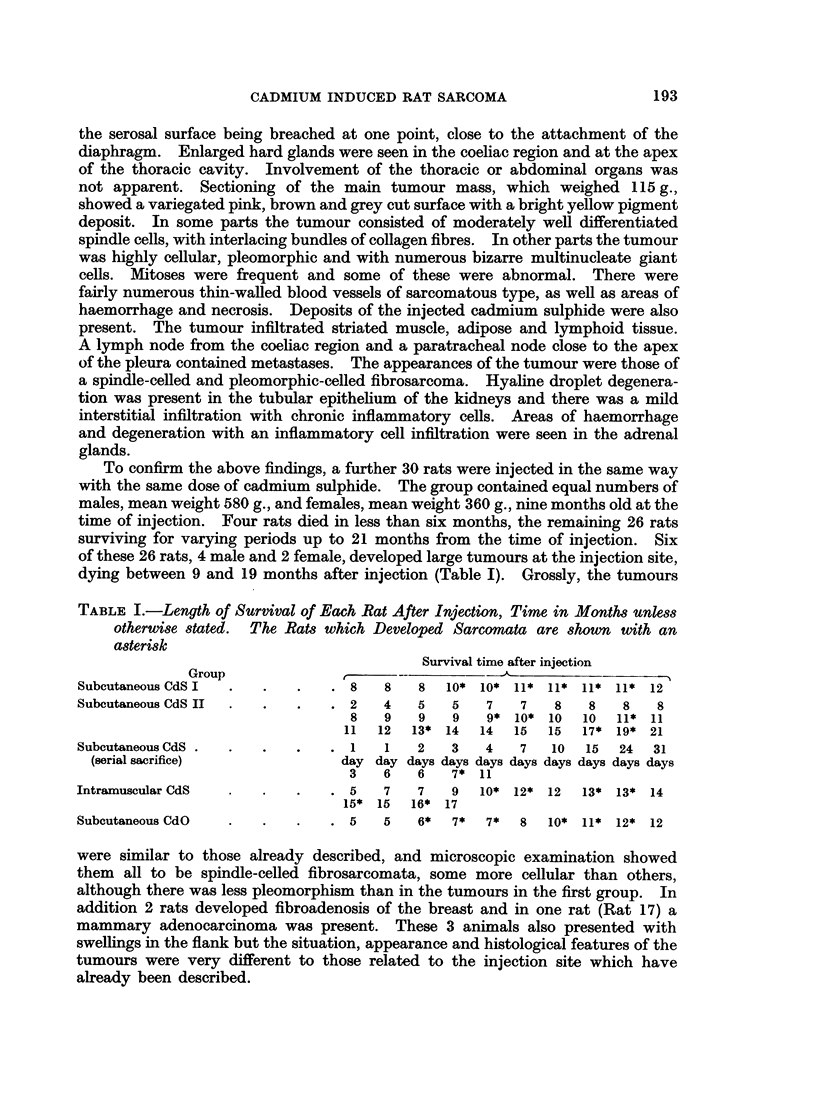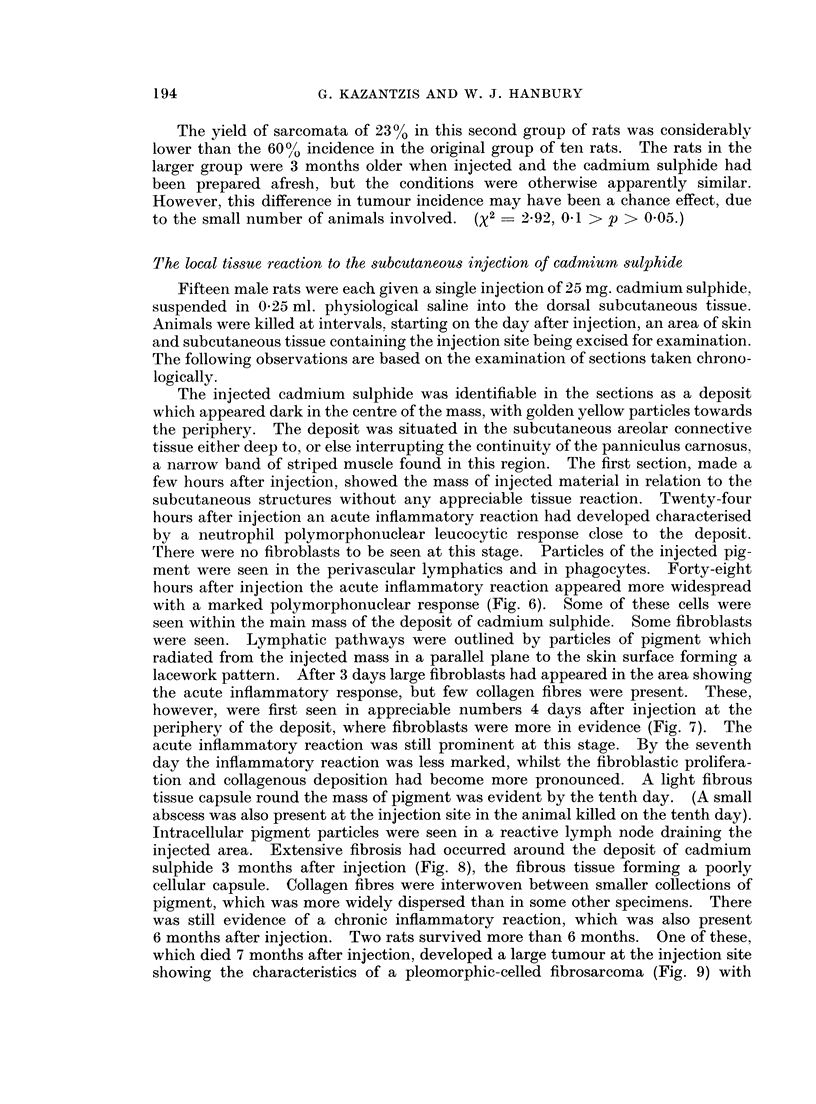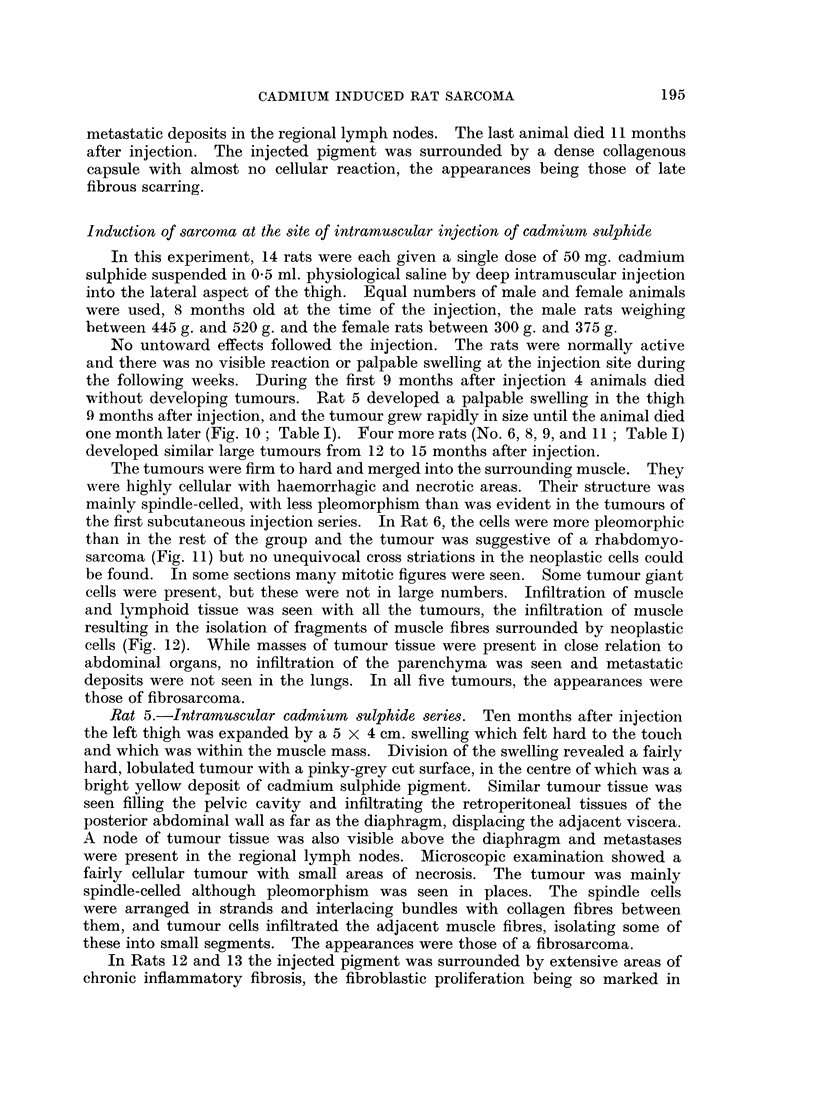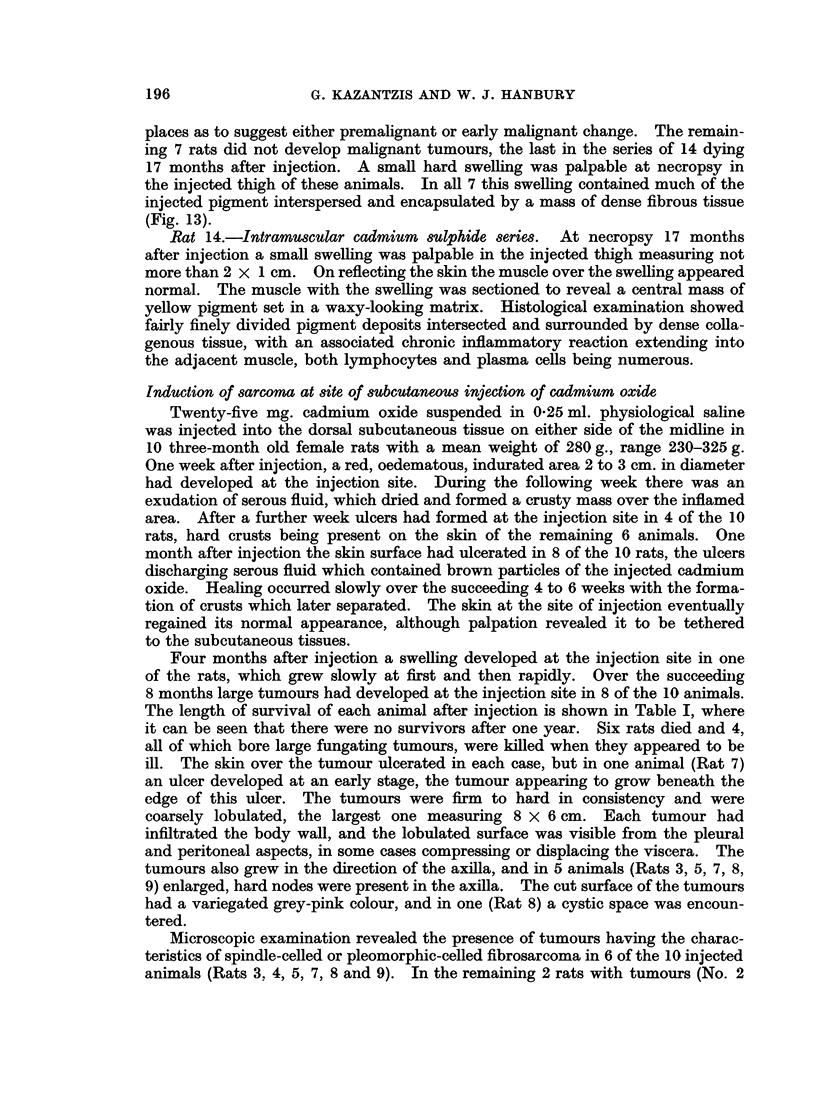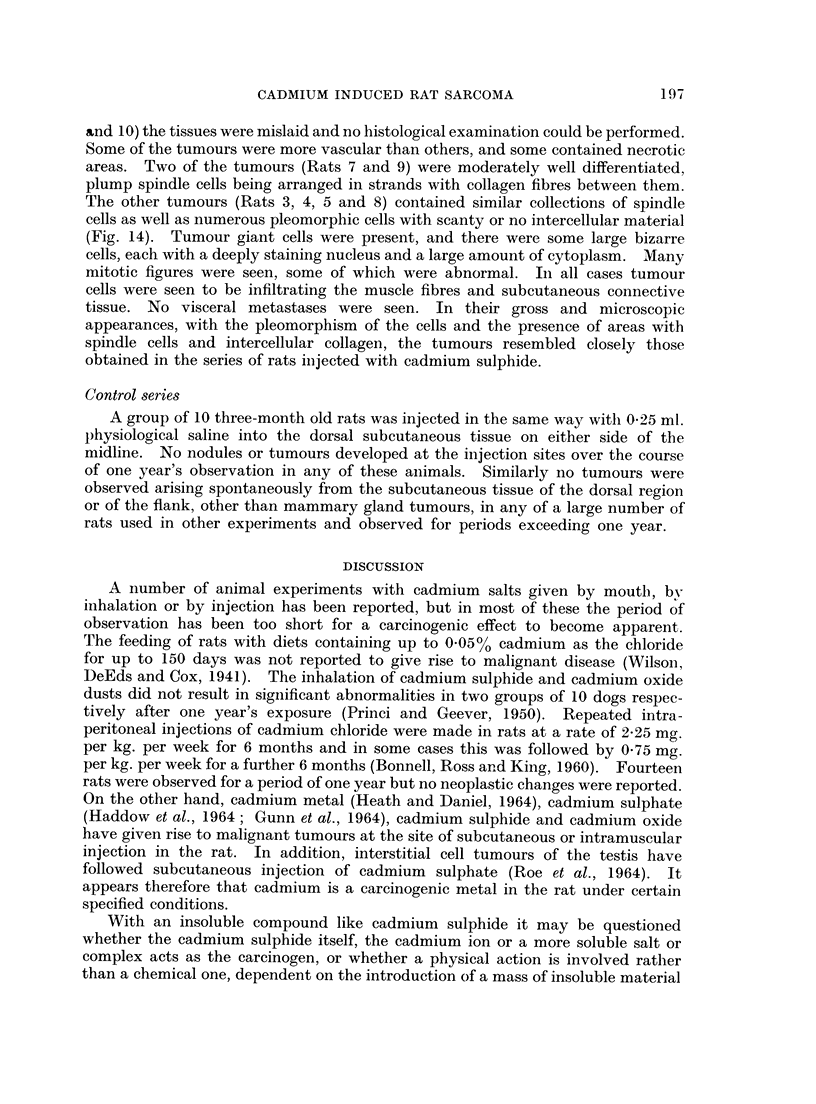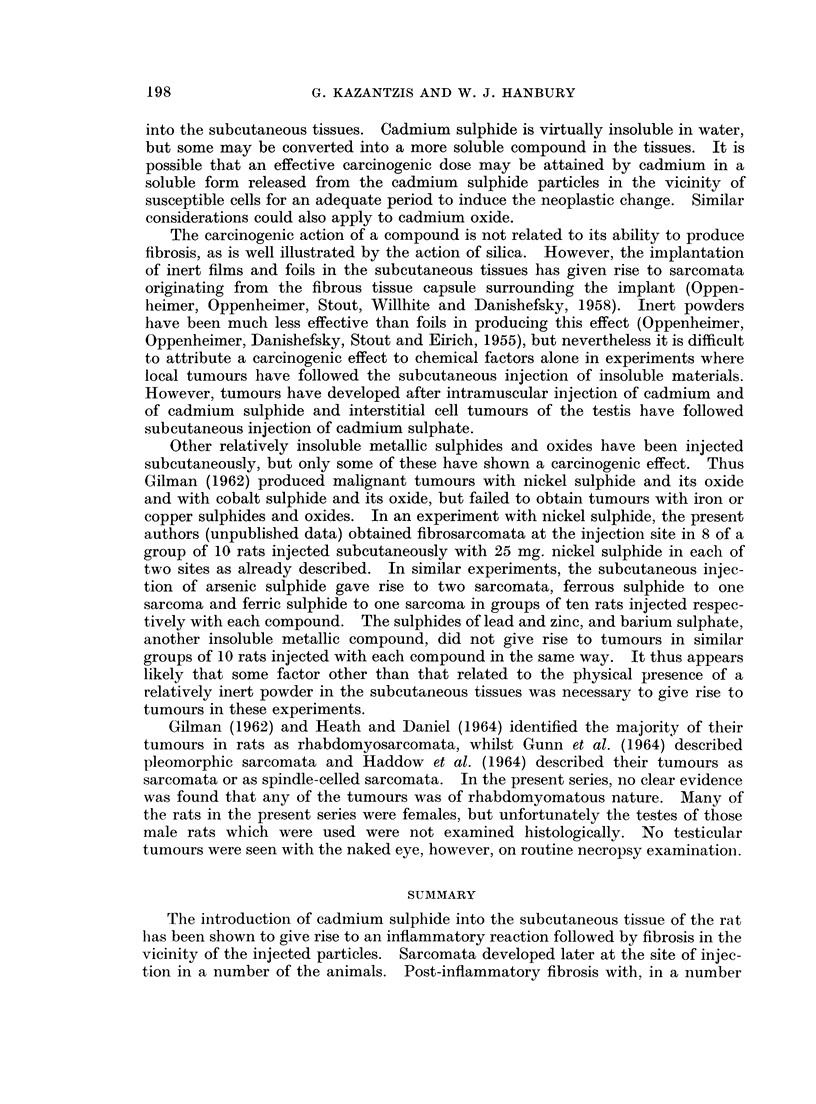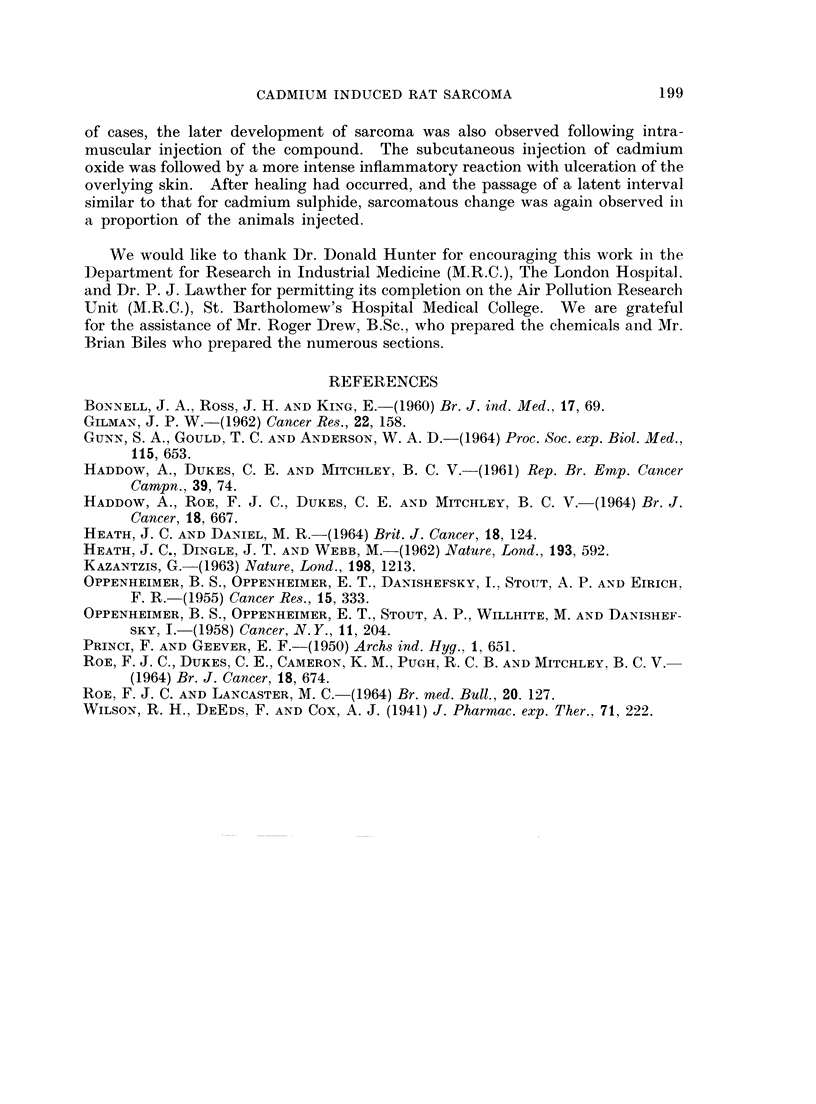# The induction of sarcoma in the rat by cadmium sulphide and by cadmium oxide.

**DOI:** 10.1038/bjc.1966.22

**Published:** 1966-03

**Authors:** G. Kazantzis, W. J. Hanbury

## Abstract

**Images:**


### THE INDUCTION OF SARCOMA IN THE RAT BY CADMIUM SULPHIDE AND BY CADMIUM OXIDE

G. KAZANTZIS* AND W. J. HANBURY

*From the Air Pollution Research Unit (M.R.C.) and the Department of Pathology, St. Bartholomew's Hospital, London, E.C.*1

Received for publication September 30, 1965

A NUMBER of metals and some of their compounds have shown evidence of carcinogenic activity in the experimental animal and a brief review of these agents has been given by Roe and Lancaster (1964). One metal currently receiving attention in this respect has been cadmium. The repeated subcutaneous injection of ferritin in a group of rats was found to produce malignant tumours at the injection site, testicular atrophy and interstitial cell tumours of the testis (Haddow, Dukes and Mitchley, 1961). The ferritin had been prepared from rat-liver protein by precipitation with a cadmium salt. Following this observation similar results were obtained in a group of rats given repeated injections of a soluble cadmium salt in the form of cadmium sulphate. (Haddow, Roe, Dukes and Mitchley, 1964 ; Roe, Dukes, Cameron, Pugh and Mitchley, 1964) Sarcomata at the injection site and testicular tumours were later reported following the single subcutaneous injection of a relatively small dose of cadmium chloride, another soluble compound (Gunn, Gould and Anderson, 1964). Finely divided cadmium metal suspended in serum and injected into the thigh muscle of the rat led to the production of rhabdomyosarcoma and fibrosarcoma in an experiment given in a preliminary report by Heath, Daniel, Dingle and Webb (1962), and described in detail by Heath and Daniel (1964). The insoluble compound cadmium sulphide has also been shown to give rise to sarcomata at the site of subcutaneous injection in the rat (Kazantzis, 1963). The results of this experiment, performed independently in 1961, together with the findings in a further series, are now given in detail. The induction of sarcomata at the site of subcutaneous injection of cadmium sulphide was confirmed, and similar tumours were shown to follow intramuscular injection. However, the intratracheal instillation of cadmium sulphide was not followed by the development of tumours, although there were other pathological changes which will be reported separately. In a further experiment a high incidence of tumours was obtained at the injection site following the subcutaneous injection of a suspension of cadmium oxide.

### MATERIALS AND METHOD

Wistar rats of the Chester Beatty strain were used. They were provided with water and with pellets of diet 41B (Medical Research Council) *ad libitum.* A standardised vitamin supplement was added, and the diet supplemented at intervals with liver, bread and milk. The animals were lightly anaesthetised with ether and the skin at the injection site was shaved and cleaned with spirit. A

*Present address: Department of Medicine, The Middlesex Hospital, London, W.1.

suspension of the cadmium compound was made in physiological saline and injected with a mantoux syringe fitted with a 20 gauge needle. The injection was made 2–3 cm. from the site of skin puncture to obviate loss of the injected material through the needle track.

Cadmium sulphide used in the experiments was prepared in the laboratory from AR grade cadmium sulphate ($3CdSO_4 . 8H_2O$) acidified with 0·25 molar hydrochloric acid, and from hydrogen sulphide, produced in a Kipp's apparatus. The precipitate of cadmium sulphide obtained in this way was filtered, washed repeatedly, dried and ground to produce a fine yellow powder. The particles of cadmium sulphide seen in tissue sections were of the order of 0·5 $\mu$ diameter, but much of the material was aggregated into larger masses. An X-ray diffraction pattern of the cadmium sulphide powder showed some defects in the crystalline structure of the material. Sulphate ion could not be detected in a sample of cadmium sulphide on testing with barium chloride.

The cadmium oxide used was GPR grade (Hopkin & Williams Ltd.). The specification of this material was cadmium oxide 99% minimum, sodium and potassium 0·05% maximum, iron 0·001% maximum, chloride 0·01% and sulphate 0·02% maximum.

Tissues for histological examination were fixed in formol saline, stained routinely with haematoxylin and eosin and, where appropriate, with Mallory's phosphotungstic acid haematoxylin and with Van Gieson's stain.

*Induction of sarcoma at the site of subcutaneous injection of cadmium sulphide*

An injection of 25 mg. cadmium sulphide suspended in 0·25 ml. physiological saline was given into the dorsal subcutaneous tissue on either side of the midline, on a single occasion, in 10 six-month old female rats weighing between 230 g. and 325 g. No ill effects appeared to follow the injection, but a soft, oedematous swelling developed at the injection site which gradually subsided after some days to leave a small, hard nodule. Six months after injection one of the nodules began to grow in size until a large tumour developed in the flank of one of the animals. Subsequently, similar large tumours developed in 5 further rats giving a total of 6 rats with growing tumours out of the group of 10 which had been injected. These 6 animals died or were killed within one year from the time of injection, when the tumours had grown to 3 to 6 cm. in diameter. Of the remaining 4 animals in the series which did not develop growing tumours, 3 died 8 months after injection and one died 12 months after injection, so that there were no survivors after one year. Table I shows the time of survival after injection and the animals which developed tumours.

The tumours were coarsely lobulated, with a firm to hard consistency. The cut surfaces were pale pink with pearly grey areas and showed foci of the bright yellow injected pigment. The tumours were growing into the body wall so that swellings were visible from the serosal surface (Fig. 1). In one case (Rat 7) the kidney had been displaced forwards by the tumour, but the peritoneal surface had not been breached. In another case (Rat 6) the abdominal wall had been penetrated and the tumour had disseminated widely over the peritoneal cavity and omentum. Axillary, posterior thoracic or abdominal lymph nodes were enlarged in all 6 animals. Histological examination showed the tumours to be highly cellular, with some poorly formed vascular spaces and areas of degeneration, haemorrhage and necrosis. In some parts, spindle cells predominated, arranged in interlacing bundles with collagen fibres between the cells (Fig. 2). In other parts the cells were large, pleomorphic and bizarre in shape with large hyperchromatic nuclei (Fig. 3). No definite evidence of cytoplasmic cross-striation was seen in the sections stained with phosphotungstic acid haematoxylin. Multinucleate giant cells were present, more numerous in some sections than in others. Many cells contained mitotic figures and many abnormal mitoses were seen where the chromatin material was fragmented and scattered in the cytoplasm. Van Gieson stained sections showed the presence of collagen (Fig. 4) which varied in amount in different tumours and in different parts of the same tumour. In some sections only fine arborisations of collagen were seen between closely packed cells. Particles which had the appearance of the injected cadmium sulphide were scattered in the tumour tissue, some being free and others intracellular. The larger clumps of pigment were clearly doubly refractile. Spindle cells and pleomorphic cells were seen infiltrating the connective tissue and muscle of the chest and abdominal wall, isolating segments of striped muscle into small islands. Metastatic deposits with cells similar in appearance to the cells in the primary tumours were seen in the regional lymph nodes (Rats 4, 5, 6, 7). The lungs in Rats 4 and 6 also contained metastases (Fig. 5). In Rat 6, deposits of tumour cells were found on the surface of the spleen, stomach and pancreas. In Rat 9, a large tumour developed in the left flank and a smaller swelling, 1 cm. in diameter at the time the animal was killed, had developed at the second injection site in the right flank. Microscopic examination of the second swelling showed a deposit of pigment surrounded by a granulomatous reaction and dense fibrosis, and in places there were plump spindle cells with irregular nuclei and mitoses.

The tumours in the 6 rats were considered to be examples of cellular, spindle-celled and pleomorphic-celled fibrosarcoma.

*Rat 5, subcutaneous CdS* (1961 *series*).—The animal was killed 10 months after injection. The skin was reflected off a large tumour on the back to expose a lobulated, hard mass, pinky grey with a yellow area, similar in colour to the injected material. The tumour bulged into the pleural and peritoneal cavities,

---

EXPLANATION OF PLATES

FIG. 1.—Subcutaneous CdS : Gross appearance of tumour.
FIG. 2.—Subcutaneous CdS : Spindle cell fibrosarcoma. H. & E. × 520.
FIG. 3.—Subcutaneous CdS : Pleomorphic sarcoma. H. & E. × 610.
FIG. 4.—Subcutaneous CdS : Sarcoma stained to show collagen. V.G. × 290.
FIG. 5.—Subcutaneous CdS : Metastatic deposit of tumour tissue in lung. H. & E. × 50.
FIG. 6.—Subcutaneous CdS : Rat killed 48 hours after injection. CdS deposit in subcutaneous tissue with associated inflammatory response. H. & E. × 75.
FIG. 7.—Subcutaneous CdS : Rat killed 4 days after injection. Acute inflammatory reaction with early fibroblastic response. H. & E. × 75.
FIG. 8.—Subcutaneous CdS : Rat killed 3 months after injection. Fibrous tissue in relation to deposited pigment. H. & E. × 75.
FIG. 9. Subcutaneous CdS : Pleomorphic sarcoma showing cells in relation to particles of pigment. H. & E. × 325.
FIG. 10.—Intramuscular CdS : Gross appearance of tumour in thigh and pelvis.
FIG. 11.—Intramuscular CdS : Pleomorphic sarcoma. H. & E. × 325.
FIG. 12. Intramuscular CdS : Tumour tissue infiltrating muscle of thigh. H. & E. × 325.
FIG. 13.—Intramuscular CdS : Fibrosis in muscle in a rat which did not develop a tumour. H. & E. × 190.
FIG. 14. Subcutaneous CdO : Pleomorphic sarcoma. H. & E. × 325.

1

2

Kazantzis and Hanbury.

3

4

5

Kazantzis and Hanbury.

6

7

8

9

10

11

12

13

14

Kanzantzis and Hanbury.

the serosal surface being breached at one point, close to the attachment of the diaphragm. Enlarged hard glands were seen in the coeliac region and at the apex of the thoracic cavity. Involvement of the thoracic or abdominal organs was not apparent. Sectioning of the main tumour mass, which weighed 115 g., showed a variegated pink, brown and grey cut surface with a bright yellow pigment deposit. In some parts the tumour consisted of moderately well differentiated spindle cells, with interlacing bundles of collagen fibres. In other parts the tumour was highly cellular, pleomorphic and with numerous bizarre multinucleate giant cells. Mitoses were frequent and some of these were abnormal. There were fairly numerous thin-walled blood vessels of sarcomatous type, as well as areas of haemorrhage and necrosis. Deposits of the injected cadmium sulphide were also present. The tumour infiltrated striated muscle, adipose and lymphoid tissue. A lymph node from the coeliac region and a paratracheal node close to the apex of the pleura contained metastases. The appearances of the tumour were those of a spindle-celled and pleomorphic-celled fibrosarcoma. Hyaline droplet degeneration was present in the tubular epithelium of the kidneys and there was a mild interstitial infiltration with chronic inflammatory cells. Areas of haemorrhage and degeneration with an inflammatory cell infiltration were seen in the adrenal glands.

To confirm the above findings, a further 30 rats were injected in the same way with the same dose of cadmium sulphide. The group contained equal numbers of males, mean weight 580 g., and females, mean weight 360 g., nine months old at the time of injection. Four rats died in less than six months, the remaining 26 rats surviving for varying periods up to 21 months from the time of injection. Six of these 26 rats, 4 male and 2 female, developed large tumours at the injection site, dying between 9 and 19 months after injection (Table I). Grossly, the tumours

TABLE I.—*Length of Survival of Each Rat After Injection, Time in Months unless otherwise stated. The Rats which Developed Sarcomata are shown with an asterisk*

| Group | Survival time after injection | | | | | | | | | |
|---|---|---|---|---|---|---|---|---|---|---|
| Subcutaneous CdS I | 8 | 8 | 8 | 10* | 10* | 11* | 11* | 11* | 11* | 12 |
| Subcutaneous CdS II | 2 | 4 | 5 | 5 | 7 | 7 | 8 | 8 | 8 | 8 |
| | 8 | 9 | 9 | 9 | 9* | 10* | 10 | 10 | 11* | 11 |
| | 11 | 12 | 13* | 14 | 14 | 15 | 15 | 17* | 19* | 21 |
| Subcutaneous CdS (serial sacrifice) | 1 day | 1 day | 2 days | 3 days | 4 days | 7 days | 10 days | 15 days | 24 days | 31 days |
| | 3 | 6 | 6 | 7* | 11 | | | | | |
| Intramuscular CdS | 5 | 7 | 7 | 9 | 10* | 12* | 12 | 13* | 13* | 14 |
| | 15* | 15 | 16* | 17 | | | | | | |
| Subcutaneous CdO | 5 | 5 | 6* | 7* | 7* | 8 | 10* | 11* | 12* | 12 |

were similar to those already described, and microscopic examination showed them all to be spindle-celled fibrosarcomata, some more cellular than others, although there was less pleomorphism than in the tumours in the first group. In addition 2 rats developed fibroadenosis of the breast and in one rat (Rat 17) a mammary adenocarcinoma was present. These 3 animals also presented with swellings in the flank but the situation, appearance and histological features of the tumours were very different to those related to the injection site which have already been described.

The yield of sarcomata of 23% in this second group of rats was considerably lower than the 60% incidence in the original group of ten rats. The rats in the larger group were 3 months older when injected and the cadmium sulphide had been prepared afresh, but the conditions were otherwise apparently similar. However, this difference in tumour incidence may have been a chance effect, due to the small number of animals involved. ($\chi^2 = 2{\cdot}92$, $0{\cdot}1 > p > 0{\cdot}05$.)

*The local tissue reaction to the subcutaneous injection of cadmium sulphide*

Fifteen male rats were each given a single injection of 25 mg. cadmium sulphide, suspended in 0·25 ml. physiological saline into the dorsal subcutaneous tissue. Animals were killed at intervals, starting on the day after injection, an area of skin and subcutaneous tissue containing the injection site being excised for examination. The following observations are based on the examination of sections taken chronologically.

The injected cadmium sulphide was identifiable in the sections as a deposit which appeared dark in the centre of the mass, with golden yellow particles towards the periphery. The deposit was situated in the subcutaneous areolar connective tissue either deep to, or else interrupting the continuity of the panniculus carnosus, a narrow band of striped muscle found in this region. The first section, made a few hours after injection, showed the mass of injected material in relation to the subcutaneous structures without any appreciable tissue reaction. Twenty-four hours after injection an acute inflammatory reaction had developed characterised by a neutrophil polymorphonuclear leucocytic response close to the deposit. There were no fibroblasts to be seen at this stage. Particles of the injected pigment were seen in the perivascular lymphatics and in phagocytes. Forty-eight hours after injection the acute inflammatory reaction appeared more widespread with a marked polymorphonuclear response (Fig. 6). Some of these cells were seen within the main mass of the deposit of cadmium sulphide. Some fibroblasts were seen. Lymphatic pathways were outlined by particles of pigment which radiated from the injected mass in a parallel plane to the skin surface forming a lacework pattern. After 3 days large fibroblasts had appeared in the area showing the acute inflammatory response, but few collagen fibres were present. These, however, were first seen in appreciable numbers 4 days after injection at the periphery of the deposit, where fibroblasts were more in evidence (Fig. 7). The acute inflammatory reaction was still prominent at this stage. By the seventh day the inflammatory reaction was less marked, whilst the fibroblastic proliferation and collagenous deposition had become more pronounced. A light fibrous tissue capsule round the mass of pigment was evident by the tenth day. (A small abscess was also present at the injection site in the animal killed on the tenth day). Intracellular pigment particles were seen in a reactive lymph node draining the injected area. Extensive fibrosis had occurred around the deposit of cadmium sulphide 3 months after injection (Fig. 8), the fibrous tissue forming a poorly cellular capsule. Collagen fibres were interwoven between smaller collections of pigment, which was more widely dispersed than in some other specimens. There was still evidence of a chronic inflammatory reaction, which was also present 6 months after injection. Two rats survived more than 6 months. One of these, which died 7 months after injection, developed a large tumour at the injection site showing the characteristics of a pleomorphic-celled fibrosarcoma (Fig. 9) with metastatic deposits in the regional lymph nodes. The last animal died 11 months after injection. The injected pigment was surrounded by a dense collagenous capsule with almost no cellular reaction, the appearances being those of late fibrous scarring.

*Induction of sarcoma at the site of intramuscular injection of cadmium sulphide*

In this experiment, 14 rats were each given a single dose of 50 mg. cadmium sulphide suspended in 0·5 ml. physiological saline by deep intramuscular injection into the lateral aspect of the thigh. Equal numbers of male and female animals were used, 8 months old at the time of the injection, the male rats weighing between 445 g. and 520 g. and the female rats between 300 g. and 375 g.

No untoward effects followed the injection. The rats were normally active and there was no visible reaction or palpable swelling at the injection site during the following weeks. During the first 9 months after injection 4 animals died without developing tumours. Rat 5 developed a palpable swelling in the thigh 9 months after injection, and the tumour grew rapidly in size until the animal died one month later (Fig. 10 ; Table I). Four more rats (No. 6, 8, 9, and 11 ; Table I) developed similar large tumours from 12 to 15 months after injection.

The tumours were firm to hard and merged into the surrounding muscle. They were highly cellular with haemorrhagic and necrotic areas. Their structure was mainly spindle-celled, with less pleomorphism than was evident in the tumours of the first subcutaneous injection series. In Rat 6, the cells were more pleomorphic than in the rest of the group and the tumour was suggestive of a rhabdomyosarcoma (Fig. 11) but no unequivocal cross striations in the neoplastic cells could be found. In some sections many mitotic figures were seen. Some tumour giant cells were present, but these were not in large numbers. Infiltration of muscle and lymphoid tissue was seen with all the tumours, the infiltration of muscle resulting in the isolation of fragments of muscle fibres surrounded by neoplastic cells (Fig. 12). While masses of tumour tissue were present in close relation to abdominal organs, no infiltration of the parenchyma was seen and metastatic deposits were not seen in the lungs. In all five tumours, the appearances were those of fibrosarcoma.

*Rat* 5.—*Intramuscular cadmium sulphide series.* Ten months after injection the left thigh was expanded by a 5 × 4 cm. swelling which felt hard to the touch and which was within the muscle mass. Division of the swelling revealed a fairly hard, lobulated tumour with a pinky-grey cut surface, in the centre of which was a bright yellow deposit of cadmium sulphide pigment. Similar tumour tissue was seen filling the pelvic cavity and infiltrating the retroperitoneal tissues of the posterior abdominal wall as far as the diaphragm, displacing the adjacent viscera. A node of tumour tissue was also visible above the diaphragm and metastases were present in the regional lymph nodes. Microscopic examination showed a fairly cellular tumour with small areas of necrosis. The tumour was mainly spindle-celled although pleomorphism was seen in places. The spindle cells were arranged in strands and interlacing bundles with collagen fibres between them, and tumour cells infiltrated the adjacent muscle fibres, isolating some of these into small segments. The appearances were those of a fibrosarcoma.

In Rats 12 and 13 the injected pigment was surrounded by extensive areas of chronic inflammatory fibrosis, the fibroblastic proliferation being so marked in places as to suggest either premalignant or early malignant change. The remaining 7 rats did not develop malignant tumours, the last in the series of 14 dying 17 months after injection. A small hard swelling was palpable at necropsy in the injected thigh of these animals. In all 7 this swelling contained much of the injected pigment interspersed and encapsulated by a mass of dense fibrous tissue (Fig. 13).

*Rat* 14.—*Intramuscular cadmium sulphide series.* At necropsy 17 months after injection a small swelling was palpable in the injected thigh measuring not more than 2 × 1 cm. On reflecting the skin the muscle over the swelling appeared normal. The muscle with the swelling was sectioned to reveal a central mass of yellow pigment set in a waxy-looking matrix. Histological examination showed fairly finely divided pigment deposits intersected and surrounded by dense collagenous tissue, with an associated chronic inflammatory reaction extending into the adjacent muscle, both lymphocytes and plasma cells being numerous.

*Induction of sarcoma at site of subcutaneous injection of cadmium oxide*

Twenty-five mg. cadmium oxide suspended in 0·25 ml. physiological saline was injected into the dorsal subcutaneous tissue on either side of the midline in 10 three-month old female rats with a mean weight of 280 g., range 230–325 g. One week after injection, a red, oedematous, indurated area 2 to 3 cm. in diameter had developed at the injection site. During the following week there was an exudation of serous fluid, which dried and formed a crusty mass over the inflamed area. After a further week ulcers had formed at the injection site in 4 of the 10 rats, hard crusts being present on the skin of the remaining 6 animals. One month after injection the skin surface had ulcerated in 8 of the 10 rats, the ulcers discharging serous fluid which contained brown particles of the injected cadmium oxide. Healing occurred slowly over the succeeding 4 to 6 weeks with the formation of crusts which later separated. The skin at the site of injection eventually regained its normal appearance, although palpation revealed it to be tethered to the subcutaneous tissues.

Four months after injection a swelling developed at the injection site in one of the rats, which grew slowly at first and then rapidly. Over the succeeding 8 months large tumours had developed at the injection site in 8 of the 10 animals. The length of survival of each animal after injection is shown in Table I, where it can be seen that there were no survivors after one year. Six rats died and 4, all of which bore large fungating tumours, were killed when they appeared to be ill. The skin over the tumour ulcerated in each case, but in one animal (Rat 7) an ulcer developed at an early stage, the tumour appearing to grow beneath the edge of this ulcer. The tumours were firm to hard in consistency and were coarsely lobulated, the largest one measuring 8 × 6 cm. Each tumour had infiltrated the body wall, and the lobulated surface was visible from the pleural and peritoneal aspects, in some cases compressing or displacing the viscera. The tumours also grew in the direction of the axilla, and in 5 animals (Rats 3, 5, 7, 8, 9) enlarged, hard nodes were present in the axilla. The cut surface of the tumours had a variegated grey-pink colour, and in one (Rat 8) a cystic space was encountered.

Microscopic examination revealed the presence of tumours having the characteristics of spindle-celled or pleomorphic-celled fibrosarcoma in 6 of the 10 injected animals (Rats 3, 4, 5, 7, 8 and 9). In the remaining 2 rats with tumours (No. 2

and 10) the tissues were mislaid and no histological examination could be performed. Some of the tumours were more vascular than others, and some contained necrotic areas. Two of the tumours (Rats 7 and 9) were moderately well differentiated, plump spindle cells being arranged in strands with collagen fibres between them. The other tumours (Rats 3, 4, 5 and 8) contained similar collections of spindle cells as well as numerous pleomorphic cells with scanty or no intercellular material (Fig. 14). Tumour giant cells were present, and there were some large bizarre cells, each with a deeply staining nucleus and a large amount of cytoplasm. Many mitotic figures were seen, some of which were abnormal. In all cases tumour cells were seen to be infiltrating the muscle fibres and subcutaneous connective tissue. No visceral metastases were seen. In their gross and microscopic appearances, with the pleomorphism of the cells and the presence of areas with spindle cells and intercellular collagen, the tumours resembled closely those obtained in the series of rats injected with cadmium sulphide.

*Control series*

A group of 10 three-month old rats was injected in the same way with 0·25 ml. physiological saline into the dorsal subcutaneous tissue on either side of the midline. No nodules or tumours developed at the injection sites over the course of one year's observation in any of these animals. Similarly no tumours were observed arising spontaneously from the subcutaneous tissue of the dorsal region or of the flank, other than mammary gland tumours, in any of a large number of rats used in other experiments and observed for periods exceeding one year.

### DISCUSSION

A number of animal experiments with cadmium salts given by mouth, by inhalation or by injection has been reported, but in most of these the period of observation has been too short for a carcinogenic effect to become apparent. The feeding of rats with diets containing up to 0·05% cadmium as the chloride for up to 150 days was not reported to give rise to malignant disease (Wilson, DeEds and Cox, 1941). The inhalation of cadmium sulphide and cadmium oxide dusts did not result in significant abnormalities in two groups of 10 dogs respectively after one year's exposure (Princi and Geever, 1950). Repeated intraperitoneal injections of cadmium chloride were made in rats at a rate of 2·25 mg. per kg. per week for 6 months and in some cases this was followed by 0·75 mg. per kg. per week for a further 6 months (Bonnell, Ross and King, 1960). Fourteen rats were observed for a period of one year but no neoplastic changes were reported. On the other hand, cadmium metal (Heath and Daniel, 1964), cadmium sulphate (Haddow *et al.*, 1964 ; Gunn *et al.*, 1964), cadmium sulphide and cadmium oxide have given rise to malignant tumours at the site of subcutaneous or intramuscular injection in the rat. In addition, interstitial cell tumours of the testis have followed subcutaneous injection of cadmium sulphate (Roe *et al.*, 1964). It appears therefore that cadmium is a carcinogenic metal in the rat under certain specified conditions.

With an insoluble compound like cadmium sulphide it may be questioned whether the cadmium sulphide itself, the cadmium ion or a more soluble salt or complex acts as the carcinogen, or whether a physical action is involved rather than a chemical one, dependent on the introduction of a mass of insoluble material into the subcutaneous tissues. Cadmium sulphide is virtually insoluble in water, but some may be converted into a more soluble compound in the tissues. It is possible that an effective carcinogenic dose may be attained by cadmium in a soluble form released from the cadmium sulphide particles in the vicinity of susceptible cells for an adequate period to induce the neoplastic change. Similar considerations could also apply to cadmium oxide.

The carcinogenic action of a compound is not related to its ability to produce fibrosis, as is well illustrated by the action of silica. However, the implantation of inert films and foils in the subcutaneous tissues has given rise to sarcomata originating from the fibrous tissue capsule surrounding the implant (Oppenheimer, Oppenheimer, Stout, Willhite and Danishefsky, 1958). Inert powders have been much less effective than foils in producing this effect (Oppenheimer, Oppenheimer, Danishefsky, Stout and Eirich, 1955), but nevertheless it is difficult to attribute a carcinogenic effect to chemical factors alone in experiments where local tumours have followed the subcutaneous injection of insoluble materials. However, tumours have developed after intramuscular injection of cadmium and of cadmium sulphide and interstitial cell tumours of the testis have followed subcutaneous injection of cadmium sulphate.

Other relatively insoluble metallic sulphides and oxides have been injected subcutaneously, but only some of these have shown a carcinogenic effect. Thus Gilman (1962) produced malignant tumours with nickel sulphide and its oxide and with cobalt sulphide and its oxide, but failed to obtain tumours with iron or copper sulphides and oxides. In an experiment with nickel sulphide, the present authors (unpublished data) obtained fibrosarcomata at the injection site in 8 of a group of 10 rats injected subcutaneously with 25 mg. nickel sulphide in each of two sites as already described. In similar experiments, the subcutaneous injection of arsenic sulphide gave rise to two sarcomata, ferrous sulphide to one sarcoma and ferric sulphide to one sarcoma in groups of ten rats injected respectively with each compound. The sulphides of lead and zinc, and barium sulphate, another insoluble metallic compound, did not give rise to tumours in similar groups of 10 rats injected with each compound in the same way. It thus appears likely that some factor other than that related to the physical presence of a relatively inert powder in the subcutaneous tissues was necessary to give rise to tumours in these experiments.

Gilman (1962) and Heath and Daniel (1964) identified the majority of their tumours in rats as rhabdomyosarcomata, whilst Gunn *et al.* (1964) described pleomorphic sarcomata and Haddow *et al.* (1964) described their tumours as sarcomata or as spindle-celled sarcomata. In the present series, no clear evidence was found that any of the tumours was of rhabdomyomatous nature. Many of the rats in the present series were females, but unfortunately the testes of those male rats which were used were not examined histologically. No testicular tumours were seen with the naked eye, however, on routine necropsy examination.

### SUMMARY

The introduction of cadmium sulphide into the subcutaneous tissue of the rat has been shown to give rise to an inflammatory reaction followed by fibrosis in the vicinity of the injected particles. Sarcomata developed later at the site of injection in a number of the animals. Post-inflammatory fibrosis with, in a number of cases, the later development of sarcoma was also observed following intramuscular injection of the compound. The subcutaneous injection of cadmium oxide was followed by a more intense inflammatory reaction with ulceration of the overlying skin. After healing had occurred, and the passage of a latent interval similar to that for cadmium sulphide, sarcomatous change was again observed in a proportion of the animals injected.

We would like to thank Dr. Donald Hunter for encouraging this work in the Department for Research in Industrial Medicine (M.R.C.), The London Hospital, and Dr. P. J. Lawther for permitting its completion on the Air Pollution Research Unit (M.R.C.), St. Bartholomew's Hospital Medical College. We are grateful for the assistance of Mr. Roger Drew, B.Sc., who prepared the chemicals and Mr. Brian Biles who prepared the numerous sections.

### REFERENCES

BONNELL, J. A., ROSS, J. H. AND KING, E.—(1960) *Br. J. ind. Med.*, **17**, 69.
GILMAN, J. P. W.—(1962) *Cancer Res.*, **22**, 158.
GUNN, S. A., GOULD, T. C. AND ANDERSON, W. A. D.—(1964) *Proc. Soc. exp. Biol. Med.*, **115**, 653.
HADDOW, A., DUKES, C. E. AND MITCHLEY, B. C. V.—(1961) *Rep. Br. Emp. Cancer Campn.*, **39**, 74.
HADDOW, A., ROE, F. J. C., DUKES, C. E. AND MITCHLEY, B. C. V.—(1964) *Br. J. Cancer*, **18**, 667.
HEATH, J. C. AND DANIEL, M. R.—(1964) *Brit. J. Cancer*, **18**, 124.
HEATH, J. C., DINGLE, J. T. AND WEBB, M.—(1962) *Nature, Lond.*, **193**, 592.
KAZANTZIS, G.—(1963) *Nature, Lond.*, **198**, 1213.
OPPENHEIMER, B. S., OPPENHEIMER, E. T., DANISHEFSKY, I., STOUT, A. P. AND EIRICH, F. R.—(1955) *Cancer Res.*, **15**, 333.
OPPENHEIMER, B. S., OPPENHEIMER, E. T., STOUT, A. P., WILLHITE, M. AND DANISHEFSKY, I.—(1958) *Cancer, N.Y.*, **11**, 204.
PRINCI, F. AND GEEVER, E. F.—(1950) *Archs ind. Hyg.*, **1**, 651.
ROE, F. J. C., DUKES, C. E., CAMERON, K. M., PUGH, R. C. B. AND MITCHLEY, B. C. V.—(1964) *Br. J. Cancer*, **18**, 674.
ROE, F. J. C. AND LANCASTER, M. C.—(1964) *Br. med. Bull.*, **20**, 127.
WILSON, R. H., DEEDS, F. AND COX, A. J. (1941) *J. Pharmac. exp. Ther.*, **71**, 222.